# Is minimally invasive parathyroid surgery an option for patients with gestational primary hyperparathyroidism?

**DOI:** 10.1186/1471-2393-13-130

**Published:** 2013-06-11

**Authors:** Cino Bendinelli, Shane Nebauer, Tuan Quach, Shaun Mcgrath, Shamasunder Acharya

**Affiliations:** 1 Department of Surgery, John Hunter Hospital, New Lambton Heights, NSW, Australia; 2 University of Newcastle, Callaghan, NSW, Australia; 3 Department of Endocrinology, John Hunter Hospital, New Lambton Heights, NSW, Australia

**Keywords:** Minimally invasive, Parathyroidectomy, Gestational primary hyperparathyroidism, Video assisted, Hypercalcemia without Sestamibi

## Abstract

**Background:**

Gestational primary hyperparathyroidism is associated with serious maternal and neonatal complications, which require prompt surgical treatment. Minimally invasive parathyroidectomy reduces pain, improves cosmesis and may achieve cure rates comparable to traditional open bilateral neck exploration. We report the clinical course of a woman with newly diagnosed gestational primary hyperparathyroidism and discuss the decision making behind the choice of video-assisted minimally invasive parathyroidectomy, amongst the other minimally invasive parathyroidectomy techniques available.

**Case presentation:**

A 38-years-old pregnant woman at 9 weeks of gestation, with severe hyperemesis and hypercalcaemia secondary to gestational primary hyperparathyroidism (ionised calcium 1.28 mmol/l) was referred for surgery. Ultrasound examination of her neck identified 2 suspicious parathyroid enlargements. In view of pregnancy, a radioisotope Sestamibi parathyroid scan was not performed. Bilateral four-gland exploration was therefore deemed necessary to guarantee cure. This was performed with video-assisted minimally invasive parathyroidectomy, which relies on a single 15 mm central incision with external retraction and endoscopic magnification, allowing bilateral neck exploration.

Surgery was performed at 23 weeks of gestation. Four glands were identified in orthotopic positions of which three had normal appearance. The fourth was a right superior parathyroid adenoma of 756 mg. Ionized calcium (1.12 mmol/l) and PTH (0.9 pmol/l) normalised postoperatively. Patient was discharged on the second postoperative day, needing no pain relief. Cosmetic result was excellent. Her pregnancy progressed normally and she delivered a healthy baby.

**Conclusion:**

Video-assisted minimally invasive parathyroidectomy allows bilateral four-gland exploration, and is an optimal technique to treat gestational primary hyperparathyroidism. This procedure removes the need for radiation exposure, reduces pain, improves cosmesis and may achieve cure rates comparable to traditional open bilateral neck exploration.

## Background

Gestational primary hyperparathyroidism (GPHPT) is a rare and often unrecognized condition [1]. Surgical treatment is the optimal and definitive treatment for GPHPT, and has been shown to reduce the incidence of life threatening maternal and foetal complications such as preeclampsia, miscarriage and hypercalcaemic crisis [1,2]. Surgery during pregnancy is indicated for all patients with GPHPT, even if hypercalcaemia is mild and there are no established complications [2,3]. Open bilateral neck exploration represents the golden standard for the treatment of primary hyperparathyroidism, though in the last decade a variety of minimally invasive parathyroidectomy (MIP) techniques have been developed. In selected patients, MIP has shown similar cure rates to open bilateral neck exploration, but with less postoperative pain, postoperative stay, overall patient distress and better cosmetic outcome [1,4-6].

We review the clinical significance of GPHPT along with the indications and timing of surgery. Additionally, we review the different MIP options available for the treatment of GPHPT, and present one patient with GPHPT successfully treated, with bilateral neck exploration performed using a minimally invasive video-assisted technique.

### Case report

A 38-years-old pregnant woman, at 9 weeks of gestation, was admitted to hospital with severe hyperemesis, thirst and polyuria. Routine investigations showed low TSH 0.04 mIU/l (0.4 – 4), mildly elevated free T4 23.4pmol/l (10.6 – 20.5), normal free T3 5.6pmol/l (3.3 – 6.2), elevated corrected calcium 2.61 mmol/l (2.17 – 2.46), elevated ionised calcium 1.28 mmol/l (1.04 – 1.24) and low phosphate 0.67 mmol/l (0.79 – 1.37). Her abnormal thyroid function was attributed to pregnancy itself and normalised without intervention. PTH was elevated 12.4pmol/l (0.8 - 8), and her vitamin D levels were subnormal 45 nmol/l (33 – 107). 24 hr urine calcium was elevated at 13.6 mmol/day (1.5-7.5), with urine volume of 1.7 litres in 24 hrs. Her previous 4 pregnancies were uneventful. She had no previous history of calcium disorders or family history of endocrine disorders. She was treated with intravenous fluids and antiemetics and was referred for dedicated parathyroid ultrasound (US) and surgical consult.

The patient was examined with US in real time (GE Healthcare Logic ® P5 with 11 MHz linear transducer) in the supine position with her neck in extension. A definite enlargement of one right parathyroid (8 × 6 × 17 mm) was identified immediately posterior to the lower pole of the right thyroid lobe, adjacent to the trachea. Another suspicious lesion of 5 × 2 × 8 mm was identified inferior to the lower pole of the left thyroid lobe.

Sestamibi scan was not requested due to its relative contraindication during pregnancy [7].

At John Hunter Hospital, a teaching and referral institution, MIP is performed either through a 20-30 mm incision and open technique, that allows targeted removal of a preoperative localized adenoma, or through a 15-20 mm incision and video-assisted technique [5,8]. Video-assisted MIP allows a complete uni- or bi-lateral exploration, and removal of one or more parathyroid tumours [9]. At our institution, intraoperative parathyroid hormone assay (iPTHa) is not available for intraoperative confirmation of complete removal of all pathologic parathyroid tissue.

In absence of concordant preoperative imaging and iPTHa, a bilateral neck exploration was deemed necessary to minimize the risk of missing multiple glands disease. Bilateral neck exploration was achieved with the video-assisted technique. The procedure was carried out using general endotracheal anaesthesia. No antibiotic was administered. A chlorhexidine based solution was utilized to achieve the sterile operative field. Video-assisted MIP technique is described in detail elsewhere [5]. Briefly, with the neck in neutral position, a 15 mm transverse incision is performed 1 cm caudal to the cricoid. Diathermy and blunt dissection allow entrance through the neck, mid-line into the plane between the strap muscles and the thyroid. Dedicated retractors (Medtronic Terris®) allow gentle traction and create the working space. A conventional, 30-degree 5 mm laparo-endoscope is utilized to allow optimal lighting and magnification. A dedicated suctioning spatula (Medtronic Terris®) allows delicate tissue dissection.

Foetal heart tones were documented with cardiotocography immediately before surgery, and then every 6 hours postoperatively for 24 hours. After parathyroidectomy, the patient was treated with oral calcium (calcium citrate 1500 mg/day) for 30 days. Serum calcium and PTH were measured 3 hours postoperatively, the following morning, and then every 4 weeks.

## Results

**Figure 1:**
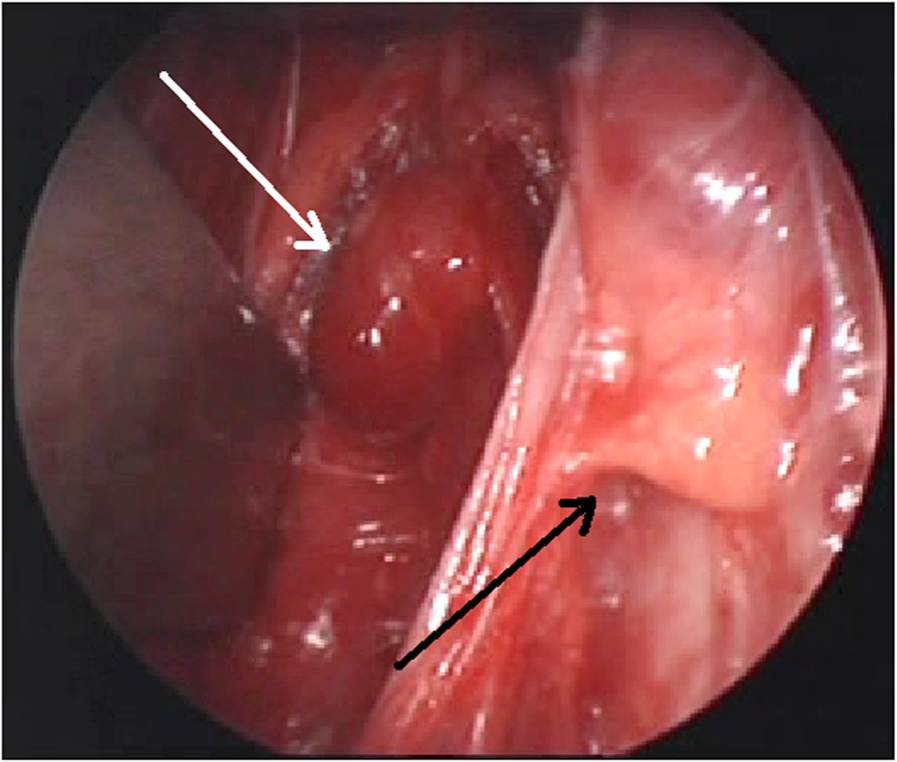
Right superior parathyroid adenoma (white arrow) and left inferior normal parathyroid gland (black arrow).

The procedure was semi-electively planned during the 23 weeks gestation. All four glands were identified in orthotopic positions. Each recurrent laryngeal nerve (RLN) was identified bilaterally. While three of the parathyroid glands had a normal appearance, the right superior parathyroid gland, lying posterior to the RLN, was a 756 mg adenoma. This was removed intact after clipping of the pedicle (Figure 1). Operative time was 80 minutes. Histology confirmed a benign parathyroid adenoma. The patient tolerated the procedure well as did the foetus, whose heart tones remained normal before, during and after the procedure.

**Figure 2:**
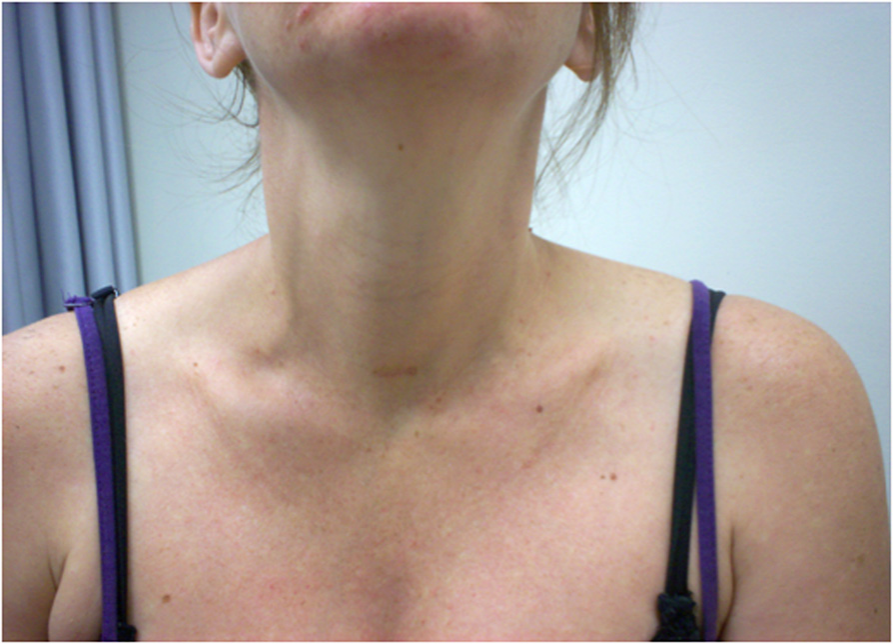
Cosmetic result at 6 weeks post surgery.

PTH levels were suppressed (<1pmol/l) immediately following surgery, resulting in normalised calcium levels (Calcium 2.38 mmol/l, ionised calcium 1.12 mmol/l). The patient experienced minimal pain and was discharged on the second postoperative day needing no pain relief. Cosmetic result was excellent (Figure 2). Her calcium and PTH levels remained normal throughout the pregnancy. The rest of her pregnancy was uneventful, and she delivered a healthy baby at 41 weeks of gestation.

## Discussion

In 1947 Petit and Clark successfully treated a patient suffering from GPHPT [10]. Under local anaesthesia and supplemented only by nitrous oxide and oxygen, they were able to remove a palpable parathyroid adenoma weighting 13 g [10]. Since then, no more than 200 similar cases have been reported [1-3]. Because the incidence of GPHPT is so low, the literature is comprised almost exclusively of case reports with few reviews and only a large series [1-3]. Its rarity accounts for missing guidelines supporting physicians in the decision making process.

The low albumin levels, typically associated with pregnancy, are responsible for the low sensitivity of serum total calcium screening tests and may explain why up to 90% of GPHPT cases are undiagnosed until after a miscarriage [1,11,12]. GPHPT can have dramatic negative effects on both mother and foetus. Maternal complications occur in up to 67% of pregnancies, with most those common being hyperemesis, polyuria and nephrolithiasis [13,14]. Untreated GPHPT may also lead to preeclampsia and miscarriage in up to 25% and 50% of cases, respectively [1,2]. More severe complications (such as pancreatitis and hypercalcaemic crisis) occur rarely. The rate of foetal complications in untreated GPHP is as high as 53%, with neonatal death occurring in up to 30% of cases [2]. Calcium is actively transported across the placenta and, when pregnancy results in a live birth, cases of profound foetal PTH suppression (with hypocalcaemia and tetany requiring intravenous calcium therapy) have been reported [15]. Permanent hypoparathyroidism and growth retardation has also been documented [15].

In non-pregnant individuals, hyperplasia and multiple adenomas occur in 10-12% and 2%, respectively. Carcinoma occurs in less then 1% of PHPT cases [16]. There are simply not enough patients reported in the literature to correctly estimate the prevalence of multiple gland disease in GPHPT. On the other hand, few cases of hyperplasia or multiple adenomas are still reported in the above-mentioned series [1-3]. The management of GPHPT is linked to peculiar problems, which we will try to discuss individually.

### Indications for surgery

The cases best managed by surgical treatment have been best demonstrated by Norman et al. [1]. A case series of 32 women (who had a total of 77 pregnancies with elevated serum calcium levels due to GPHPT) showed that even a mild elevation of calcium (as moderate as 2.67 mmol/l) was associated with a 12% risk of pregnancy loss [1]. Parathyroidectomy is indicated for all GPHPT patients, with or without symptoms, even in mild hypercalcaemia [1-3].

### Timing of surgery

Surgical intervention in general, and parathyroidectomy specifically, has always been considered more acceptable during the second trimester [3]. According to the largest series of patients with GPHPT, the majority of pregnancy losses occur during the late first or early second trimester. The Authors therefore recommend to time parathyroidectomy in the late first trimester (instead of waiting until the mid-second trimester) [1]. Even in case of a delayed diagnosis of GPHPT, surgery should not be delayed any further, nor performed after delivery [2]. The increased foetal and maternal risks of delayed parathyroidectomy compare negatively with the most common and easily treatable postoperative complications (namely hypocalcaemia, which happens in up to 60% of patients following bilateral neck exploration) [2]. Given the favourable benefit-to-risk ratio, parathyroidectomy should be performed as early as possible and, in case of late diagnosis, even during the third trimester [1-3].

### Preoperative imaging

Accurate preoperative localization of parathyroid adenomas allows the targeting of suspicious glands and has sparked the development of MIP techniques. The most commonly employed preoperative imaging techniques are parathyroid radionuclide imaging (Sestamibi) and US.

Sensitivities of Sestamibi vary widely from 39% to over 90%. The discrepancies reported relate to both differences in technique and patient cohorts, as Sestamibi uptake is influenced by PTH levels and the presence, or absence, of thyroid nodules [17]. Unfortunately during pregnancy, Sestamibi is not an ideal option due to the risks linked to foetal irradiation. Sestamibi should be reserved only for patients with persistent GPHPT following non-curative cervical explorations, as recently reported on a series of 7 patients [7].

In experienced and dedicated hands, high frequency US is a highly sensitive technique (sensitivity of 89% and PPV 98%); especially in patients without thyroid nodules [17]. The absence of ionizing radiation explains why US is the most commonly used diagnostic tool for many pathologies during pregnancy. The primary limitation of US resides on the fact that optimal results are exquisitely operator dependent and not easily reproducible in less experienced hands [18].

Although computed tomography (CT) scanners use high doses of ionizing radiation, the foetus can be efficiently screened. Despite newer equipment and algorithms (such as four-dimensional CT) that improve overall parathyroid adenoma detection rate, CT sensitivity and specificity remain lower than either Sestamibi or US [17,19]. Similar considerations apply to magnetic resonance tomography, which although a promising option (especially when fused with Sestamibi), lacks sensitivity especially for smaller adenomas [17]. Venous sampling is recommended only before revision surgery [17].

In practical terms the only preoperative localization study suitable for GPHPT patients is a dedicated US.

### Anaesthetic considerations

Like the case reported here, the majority of thyroid and parathyroid surgery performed today is carried out under general anaesthesia [20]. Alternatively, local anaesthetic use in thyroid surgery has been described and recommended by many authors [20-22]. Spanknebel and colleagues reported on a series of 1,025 patients who underwent regional C2-C4 superficial cervical and local field block for thyroidectomy by the same surgeon, at the same major tertiary referral center, over 16 years [21]. They concluded that performing thyroidectomy using local anaesthesia appeared safe for most patients, including those who had a general anaesthetic risk or required more complex procedures, when performed by an experienced surgeon.

Of the 1,025 patients who received local anaesthesia, 3.3% (34) had to be converted to a general anaesthetic during the procedure. Two-thirds of these patients had either sub-sternal goiter or extensive cancer, hence the authors recommend careful selection of patients for local anaesthesia with the caveat that regardless of the requirement to convert, they were done so safely, both electively and urgently.

Thyroidectomy under local anaesthesia, specifically using a minimally invasive video-assisted technique, has been reported by Lombardi et al. [22]. Five cases were described. There were no reported conversions to either general anesthesia or to conventional surgery; neither were there postoperative complications. All of the patients were reported to be satisfied with both their surgical experience and cosmetic outcomes.

### Choice of surgical technique

MIP techniques have proven in randomized controlled trials to reduce pain, improve cosmesis and achieve a similar cure rate when compared to the golden standard: open bilateral neck exploration with identification of the four parathyroid glands [4-6,23]. Different minimally invasive techniques have been described to remove a preoperatively well-localized parathyroid adenoma. These are the options:

1) Open focused parathyroidectomy, through a 20-30 mm incision performed over the adenoma, is an option favoured by many [8,24-26]. This is a logistically simple and straightforward procedure that uses open surgery instruments to remove the localized adenoma. It is recommended for cases with concordant imaging, or at least with a positive Sestamibi scan. To ensure complete removal of pathologic tissue, the use of iPTHa is recommended by many but not all authors, especially in case of concordant localization [24,27,28]. Norman used intraoperative nuclear mapping, which is not an option during pregnancy [25]. The main limitations of this technique are: longer scar, poor visualization of RLN, limited (if not impossible) visualization of the ipsilateral parathyroid gland and above all, missing a further pathologic parathyroid gland [4,7].

2) Fully endoscopic procedures rely on neck insufflation with CO2 and 3 mini-trocars. The procedure is technically challenging, it risks spreading CO2 through tissues and increasing carbonaemia, but it allows exploration of both ipsilateral parathyroid glands and a clear visualization of the RLN. On the other hand it requires replacement of all the 3 trocars to explore the contralateral side [29].

3) Video-assisted MIP is definitely the most versatile MIP technique. It relies on external retractors, endoscopic magnification and dedicated spatulas. Through a single 15 mm incision it allows bilateral neck exploration, total or subtotal parathyroidectomy, and total or hemi-thyroidectomy [9,30,31]. Each RLN is typically well and easily localised and does not require neck insufflation. Despite being technically more challenging than open focused MIP, the logistics are quite simple as all is needed is the usual endoscopic towers, a conventional 30 degree 5-mm laparo-endoscope and a few non-disposable instruments.

4) Other fully endoscopic techniques have been described that use periareolar and axillary ports. These involve large subcutaneous dissection and gas insufflation. These latest techniques may offer some cosmetic advantage, but are technically and logistically very challenging. Most importantly, due to the large dissections they cannot be considered minimally invasive options [32].

When dealing with GPHPT, the importance of achieving cure in a timely and efficient fashion cannot be overemphasized. Traditional bilateral neck exploration achieves a cure rate of at least 95%. To guarantee comparable cure rates with MIP, the latest propositional statements recommend the use of iPTHa during targeted parathyroidectomy for patients with a single (or two non concordant) preoperative localisation study [27]. When preoperative Sestamibi and US localisation do concord for single-gland disease, iPTHa may not be necessary [24]. Following that, Sestamibi is not a safe preoperative localisation option in pregnancy; the use of iPTHa is therefore recommended to safely perform a targeted MIP for GPHPT. As iPTHa was not available to us (as is the case in many institutions), we offered to our patient the only MIP option that allows bilateral 4-gland exploration: the video-assisted MIP.

## Conclusion

GPHPT is rare, often underestimated and best treated with surgery. When iPTHa is not available, video-assisted MIP with bilateral neck exploration should be recommended to minimize the risk of persistent disease to both mother and child. This case report illustrates the successful surgical treatment of GPHPT with a minimally invasive technique that does not require radionuclide preoperative imaging.

### Consent

This study receive ethical waiver from the local ethical authority. Informed consent was obtained from the patient for procedure and publication.

## Abbreviations

GPHPT: Gestational primary hyperparathyroidism; iPTHa: Intraoperative parathyroid hormone assay; MIP: Minimally invasive parathyroidectomy; PHPT: Primary hyperparathyroidism; RLN: Recurrent laryngeal nerve.

## Competing interests

The authors declare that they have no competing interests.

## Authors’ contributions

Study conception and design: CB, Acquisition of data: CB, SN, SM, Analysis and interpretation of data: CB, TQ, SM, SA, Drafting of manuscript:CB, Critical revision of manuscript: SN. All authors have read and approved the final manuscript.

## Pre-publication history

The pre-publication history for this paper can be accessed here:

http://www.biomedcentral.com/1471-2393/13/130/prepub
